# Magitot on the Development and Structure of the Human Teeth

**Published:** 1859-07

**Authors:** 


### AMERICAN JOURNAL

OF

### DENTAL SCIENCE.

Vol. IX. NEW SERIES—JULY, 1859. No. 3.

### ORIGINAL COMMUNICATIONS.

### ARTICLE I.

*Magitot on the Development and Structure of the Human Teeth.* Translated for the American Journal of Dental Science.

(Continued from No. 2, April, 1859.)

#### HISTORICAL.

The mode of development of the ivory, always considered very difficult to elucidate, is one of the subjects which have attracted the attention of authors, and elicited much discussion and research. At this day anatomists are yet divided on this interesting question, and it is difficult to foresee when they will agree. The microscope, it is true, has already done justice to the ancient theories, and if, at this time, this precious instrument meets serious adversaries in France, there seems to be no doubt, however, that under the eye of the anatomist who sincerely studies facts that he may draw rigorous deductions, it will eventually afford the complete solution of the important problem which at present occupies us The question has still made great progress, and we are now far removed from the old ideas, which, considering the tooth a bone, would also form a theory of dentification. The doctrine of Rau, Hunter, and Cuvier, which reversed the ossification notions, has been itself long since shaken in England and Germany by the progress of microscopic anatomy; but with us, where this science has only met with defiance and incredulity, the question of odontogenesis has remained stationary since the ideas of Hunter, revived by Cuvier, were announced, and around which the school of our day yet rallies the greatest number of anatomists.

We find in the history of odontogenesis four principal doctrines, which we are going to study, successively, to wit:

1st. The doctrine of ossification, or the ancient doctrine, which considers the tooth as a bone, and its formation as a variable ossification.

2nd. The doctrine of secretion, which regards the tooth as the result of an exudation of the pulp, a secreted product, and, according to some authors, inorganic.

3rd. The doctrine of conversion, which supposes that the tooth is formed at the expense of the pulp by a series of transformations of its tissue. This third doctrine differs essentially from the first, with which all the authors confound it, in that they do not consider the tooth as a bone, but as a particular organ of nature, very different from the bone in its structure and mode of development. Among the writers who defend this doctrine, some consider the ivory as resulting from a transformation of only one part of the pulp—the superficial part—while others believe that the totality of the organ is concerned in this formation.

4th. Finally, we have the doctrine of Raschkow, modified in England by Huxley from whom it has received the name of *theory of deposition.* It considers the ivory and the enamel as produced by special elements, strangers by themselves to the germs which give them birth. We shall adopt the fundamental idea of this doctrine which we propose to designate by the term of doctrine of the development by *autogenesis*, or the doctrine of *autogenesis*, (from αυτου, and γενεσις,) the anatomical elements of the tooth develop spontaneously, according to us, (autogenetic generation) from the surface of the germs without direct participation of their tissue.

If we have been able to formularise this doctrine in a scientific and positive manner, we owe it to alternate observation and to the profound theory of the differences which separate constituents from products; we also owe it partially to a precise knowledge of the different modes of birth of the anatomical elements; to the notion, in a word, of the differences which separate the *genesis* of the non-existing element from its *reproduction* by the aid of pre-existent elements, as this is effected, it is true, by certain cells. All these considerations, inspired moreover, for the most part from the lessons and works of our master M. Ch. Robin, will be developed hereafter.

The doctrines which we will pass in review on the subject of the development of the ivory, could be applied for the most part to the development of the enamel; perhaps the reproach may be cast upon us that we have not united in the same chapter, the history of the formation of the enamel and that of the dentine. We have made this distinction however, to preserve a clearness and precision which we should not be able to do were we to examine simultaneously those two questions. We should say in advance that writers have ofen applied an identical theory to the two phenomena. Thus the doctrine of *ossification* regards the ivory and the enamel as two osseous substances; that of *secretion* considers them as secreted products; that of *conversion*, as the result of organic transformation ; that of *deposition* itself assigns a similar law of origin to these two tissues. We will see that in fact, this analogy, admitted oftener, *a priori*, receives the sanction of microscopic observation. Nevertheless we persist in separating the two *histories*, reserving to ourselves to mention hereafter the particular facts which relate to the subject of the development of the enamel.

### 1. *Doctrine of Ossification.*

The doctrine of ossification carries us back to the earliest periods of science, and it reigned unassailed until Hunter's time. Based upon the analogies of external aspect and chemical composition which exists between the tooth and osseous tissue, it was able to satisfy the mind at a time when ostogenesis had as yet been completely ignored ; so that if we now elevate ancient notions to the dignity of a doctrine, it is because writers of note have endeavored recently to revive them, and reduce them to reason and observation.

Volcher Coiten * seems to be the first who, struck by the circumstances that the dental pulp diminished in proportion to the formation of the ivory, and still governed by the conviction of its osseous nature, admitted that the dentine, to which he gave the name of osseous part of the teeth, is but the result of veritable ossification of the germ. This sort of consecration, wrought by science, of a theory till then vague and without support, remitted it for a long time in vigor, to an epoch when the correctness of old ideas would soon be doubted. During 1683, in fact, Leeuwenhoeck † clearly laid down the differences of all the characteristics which separate the osseous tissue from the dental, and it seems that the remarks of this writer, based upon observation of facts, should ruin the theory of ossification. The successors of this anatomist who followed the new voice, abandoned, it is true, the old

Corp. part. tabula, 1573.

† Microscopical Observations on the Structure of Teeth and other Bones, (Philosophical Transactions, 1683, p. 1002.)

errors; but their numbers were, for a long time, limited and the report of the new ideas did not make the circle of the several universities. It is this which explains the long period of inaction which separated the first efforts, (1683) from the modern epoch when the studies of Leeuwenhoeck were revived by Owen and Nasmyth (1839.) However, in the camp of micrographers some authorities have recently stood forth in favor of the theory of ossification. Thus Henle in 1838,* seeking to re-establish the parallel between bone and the teeth, as far as admitting a resemblance between the dental germ and the cartilage of ossification, and starting from this principle, he concludes that dentification is a true ossification, the dental tissue a true bone. He admits a difference, however, between these two phenomena, which consists in this, that in ossification the calcareous materials are deposited in the interior of the cartilage, whilst in the dentification they are deposited on the surface of the germ and gradually invade the mass of the organ.

Bichat, in France, from the example of the principal anatomists who preceded him, has defended an analogous doctrine,† placing the description of the teeth in the study of the osseous system, and considering ivory as a bone to which he attributes a density like that of rock, he comes to explain the metamorphosis of the bulb into dentine by the phenomenon of true ossification. But, if the ancient doctrine, attacked by Rau in Germany, Hunter in England, and Cuvier in France, finds an echo in the works of the great physiologist, we must attribute it to ignorance of microscopic researches, which remain with us, and which were already in his day prolific in results. But now, thanks to a powerful instrument, some light has been and will be further thrown upon this difficult question; the progress of general anatomy does not permit us to place the teeth and bones in the same group, nor even to authorize

* Anatomie gênerale, (translated into French by Jourdan,) t. 1, p. 444, da Systemat. (Beschreiburg der Plagiostomen, 1838.)

† Anatomie generale, edit. Encyclopid., t. iii, p., 281.

their modes of formation to be attributed to the same phenomenon, yet notwithstanding, it seems that a powerful voice has come to defend anew the ancient doctrine. However, M. Flourens* has undertaken at several periods, to re-establish it among us. For him, odontogenesis is a very simple thing; "the bulb produces the tooth, by a series of transformations, as the periosteum produces bone; the bulb is transformed into cartilage, and the cartilage into bone. The bulb and the ivory are the same substance, the same lamella; there gelatinous, here penetrated by calcareous phosphate, hard, ossified; and the lamella are furnished with vessels and nerves."† Thus then for the illustrious academician, the theory of the formation of ivory would be led back to the old ideas, and this tissue would be provided with vessels and nerves. This opinion does not seem to us at present to be discussable and we must permit ourselves to regret that M. Flourens should allow his great name and authority to support a doctrine so justly fallen into oblivion. One knows the arguments invoked by modern anatomists against the identity of osseous and dental tissues, arguments drawn from the situation, from the structure, from the mode of development, and from the functional conditions which characterise these two substances.‡ Chemical analysis itself indicates the differences and constitutes another argument. To these characteristics, M. Oudet|| has offered another which plays an important part in philosophic anatomy; it is the place which the teeth occupy in the scale of vertebrata as dependences of the tegumentary system. This is regarded by the learned dentist as one of the most powerful arguments in favor of the doctrine of secretion, but we will see further that if this is confirmed, it furnishes proof against the theory of ossification, to which it cannot

* Recherches sur le developpement des os et de dents (Paris, 1842,) et Cours de Physiologie, 1855.

† Cours de Physiologie l'union Medicale, 1855, p. 499.

‡ Cruveilhein *Anat. descript.*, t. i, p. 502.

|| *Odontogenie*, p. 16, from the *Bulletin de l'Academie de Medicine*, t. xx, p. 534.

give assistance as a doctrine, already fallen before the power of observation.

2. *Doctrine of Secretion.*

The first idea of the formation of ivory by secretion or transudation should be attributed to Rau.* According to this anatomist, the dental pulp has a structure analogous to the membrane which lines the frontal sinuses ; it is composed of folds between which the secretion of a particular liquid, called the *dentific liquor*, by successive transformations, forms the layers of ivory. This liquor, deposited from the surface of the dental pulp, moulds itself upon it, augments gradually in density, and constitutes at last the dental tissue. As to the formation of the enamel, it results, according to this writer, from a secretion of another nature, but still under the agency of the pulp.

This doctrine seems to have been received with little favor in Germany, but in England owing to the impulse given to the study by Hunter's works, it met with the consecration of public favor. The illustrious anatomist himself, however, abstained from accepting Rau's propositions ; his experience demonstrated to him that ivory had no vessels, and that its development, according to the results of his experiments in feeding certain animals with madder,† could not be considered like that of a bone, but he refused to pronounce upon its true nature, which seemed to him very different from the organization of other tissues of the economy, since he concludes by saying that the teeth "should be regarded as foreign bodies in consideration of the absence of all circulation in them."‡

He approaches the development of the enamel with less reserve: "The enamel," says he, "appears to be secreted

* *Disputatio inauguralis de ortu et regeneratione dentium.* Leyde, 1694.

† For an account of these experiments see Hunter on the Teeth, vol. 1, old series of the American Journal and Library of Dental Science. Also Carpenter's Human Physiology, on the Development of Bones.—*Tr.*

‡ *Histoire Naturelle des Dents*, 1771 ; French translation of Richelot, p. 42.

from the pulp above described, and perhaps from the capsule which encloses the body of the tooth. That it is from the pulp and capsule seems evident, in the horse, the ass, the ox and the sheep, etc., therefore, we have little reason to doubt of it in the human species. It is a calcareous earth, probably dissolved in the juices of our body, and thrown out from these parts, which act here as a gland. After it is secreted, the earth is attracted by the bony part of the tooth, which is already formed ; and upon that surface it crystallizes. The operation is similar to the formation of the shell of an egg, the stone in the kidneys and bladder, and the gall stone.*

Hunter, we perceive, expresses himself with sufficient formality. According to him, the tooth is a foreign body, and if the nature of ivory escaped him, that of the enamel placed this tissue among the secreted and crystallized, or that is to say—inorganic products.

Such are the views which, notwithstanding the efforts of micrographers, have reigned for a long time in England, where, however, they are now completely abandoned; but in France, where the name of Hunter had already been heard before the beginning of this century, they have been revived by Cuvier,† who says, that the whole tooth is the result of a secretion, the ivory produced by the pulp, and the enamel by the capsule. Since then, all anatomists have been unanimous in sentiment, and the doctrine of secretion has been established among us. It reigns to day in the schools, where powerful voices propagate and defend it. Among the more serious defenders, we may cite F. Cuvier, De Blainville, E. Geoffroy Saint Hilaire, Blandin, MM. Serres, E. Rousseau, Duvernoy, Dujardin and Oudet. Such names will suffice to show the empire that Cuvier's theory yet controls and how difficult it will be to combat its influence.

* Loc. cit., p. 66.

† *Ossements Fossiles,* 4th ed., t. i, p. 510, and *Leçons d'Anat. Comp.,* 2d ed.

According to F. Cuvier,* teeth, nails, hair and horns are substances secreted by bulbs which cover them and constitute, by consequence for him, bodies foreign to the organism. The tooth, nevertheless, thinks he, may be assimilated to bone, of which the vessels, united into only one mass, may deposit calcareous matter around them; whilst bones can be regarded similarly to teeth in the interior of which, vessels, after some fashion, circulate this matter. One may easily comprehend, as has been remarked by M. Oudet, to what errors such a theory conduct in the voice of hypothesis and of induction.

De Blainville, in his numerous works, has greatly developed the doctrine of secretion, which in their turn have established his true philosophic character. Founding his theory of the *phanères* in 1822,† he first conceived the great idea of considering the teeth as dependencies of the tegumentary system; but he did not stop there, and the phanère became at last, in his eyes, an inorganic substance, a dead part excreted by a living bulb, vascular and sensitive. Such was the tooth, a true inert body, a stranger to the vital movement, and unprovided, after its formation, with any organic modifications. We will see that this is incorrect, and the only arguments which we shall oppose to the ideas so learnedly developed, will be drawn from observation with all the value which can be derived from them and the facts already studied by most writers, and which we hope will prove incontestible. We are far, however, from pretending to attack the great determination of *phanères* from a morphological point of view, but we hope to prove that the tooth, a *phanère* which we have particularly studied, is formed by elements not excreted by the bulb, but created spontaneously from its surface; and whatever may be the separation between our ideas and those of the great number of modern writers,

† *Dents des Mammifères,* t. iv, p. 214. P. 20, 1822.
‡ *Organisation des Animaux,* t. i, p. 37, 1822.

we cannot but defend our own with a certain degree of confidence, based uniquely upon the authority of observed facts. We shall develop the remainder of these considerations a little further on, but we can safely say in advance, the *phanères* studied in general by our master, M. Ch. Robin, are all united under the same mode of development with other tissues, that is, the genesis or spontaneous generation, and that they are entirely foreign to all species of phenomena of secretion.

E. Geoffroy Saint Hilaire* goes further still than his predecessors, and develops an idea already emitted by Hunter; he regards the tooth as a crystallized product, analogous to stalactite. Without discussing them we will give his own words:

"The tooth is a product of transudation, an inorganic body, a mass composed of successive layers which we are not able to compare to anything else than osseous tissue, if this is not at first a cartilaginous substance, (p. 23.) The particles of the dental substance traverse the covering of the dental nucleus by a transudation exercised from within outward. The molecular substances then unite by *crystallization.* I do not intend to speak figuratively; the work is the same; it has the same vitreous fracture, the same mode of reunion." (P. 25.)

M. Duvernoy, in his last memoir upon the teeth of musaraigns, seems to be shaken by the works of Richard Owen and Nasmyth, and makes, so to speak, a concession to the doctrine of conversion of which the two anatomists are the most eminent defenders; he offers a theory which seems to occupy a middle place between that of Cuvier and the theory resulting from microscopic investigation. According to him, the dental bulb is composed of two distinct parts, each intended for a particular function; although it may be very difficult, adds he, to determine the limits which separate them. One of them, in relation to the

* *Système Dentaire des Mammiferes et des Oiseaux;* Pairs, 1824.

blood vessels which arrive at the capsule, would be a kind of follicle to the secreting wall, which empties into the cavity which it constitutes the elements of the tubulous substance, (ivory:) it is for the time, the preparatory organ, and the reservoir of these materials. The other part of the bulb which envelops the first, and is in contact with the walls of the cavity which encloses it, composes the origin of the membranous tubes which make the canvas of the ivory, and which the materials secreted by the central part are destined to transform. (Loc. cit. p. 82.)

It cannot be denied that this opinion offers much that is vague and obscure; we cannot well seize, for example, that which concerns the dental follicle in man, that division of the bulb into two parts, the one secreting, the other destined to impregnate the calcareous materials produced by the first.

That which M. Duvernoy calls the origin of the membranous tubes of ivory, are without doubt the cells of dentine ranged on the surface of the bulb; the following phrase seems at least to indicate it. "We see the membranous tubes formed like a ring about the bulb, when we prepare a section in a bulb of the incisor of a rodent where this organ is always in activity." (Loc. cit., p. 84.)*

Be that as it may, this theory seems to want clearness and precision. That of Cuvier and his successors, MM. Serres, E. Rousseau, etc., is more precise; the bulb secretes the ivory, which is placed as a cap upon the forming organ, whilst the internal membrane of the follicle secretes the enamel, which is deposited and fixed to the surface of the ivory. Such is the simple theory, which having led Purkinje and Frœkel† into error, has been sustained in Germany; Retzius‡ and Muller§ have also

* *Memoire sur tes Dents les Musaraignes;* Paris, 1844.

† De Penitiori Dentium Humanuorum Structura Observationes; Bratislav., 1835.

‡ Bemerkungen über den innern Bau der Zähne. Mull. Arch., 1839.

§ Poggendorff's Annalen, t. xxxviii, and Cours de Physiologie, t. ii, p. 317, French trans.

leaned upon it in their works. These writers, resting upon the formation of the teeth by juxta-imposed lamella, suppose that these are excreted by the bulb. Muller made, however, some exceptions in favor of several fishes, the teeth of which, says he, are the result of the transformation of the pulp.

Finally, we come to M. Oudet, the last in chronological order, who acts as champion for the doctrine of secretion. He has in all of his writings, vehemently opposed the adverse doctrines. Guided by induction and comparative anatomy, the two means of investigation which find with him the most favor, rejecting the results owed to the intervention of the microscope, and the great authorities of Germany and England, the learned dentist defends with lively ardor, a violent theory which has been long since attacked in both countries, but which yet lingers in France, where odontogenesis seems worn out. Attributing to the teeth the place they occupy in the productions of the tegumentary system, he does not hesitate to say that not only does he "decide also the nature of their organism, but he imposes on them besides, the anatomical and physiological characteristics of the tissue to which they are united."* We are allowable in saying that here and there is a grave abuse of induction. However, M. Oudet concludes that the teeth cannot be considered as the result of the transformation of the dental pulp, the surface of which, as he has remarked, does not afford any adherance with the primitive layers of ivory, but that these organs result from an aggregation of particular materials secreted on the surface of the germ. Refusing to address himself to the teeth of man, "the complicated composition of which hides the true character of the organic acts which form them and render them deceitful:"† he asks of rodents the secret of the development of the teeth. Now what do we find in these animals? The continued growth of the tooth

*** Odontogénie, p. 9.**

**† Loc. cit., p. 10.**

with preservation and augmentation of the volume of the organ. Do we see in this phenomenon an argument in favor of the doctrine of secretion? By no means. One finds, it is true, in this case, a very remarkable particular of the development of the teeth ; a particular which consists in this, that the production of the cells of dentine is permanent in these animals, and endowed with great energy, at the same time that the volume of the pulp remains stationary or grows gradually in order to assist in this production, and also to resist the injury caused in the crowns by triturations. But in the presence of the structure so clear and uniform, which presents the teeth of rodents like those of man ; in the presence of functional acts which these organs accomplish ; in the presence of organic alterations which offer, and finally, leaning upon microscopic observation of the teeth, we do not think the doctrine of secretion able to sustain itself. We hope that M. Oudet may pardon us for the radical opposition with which we have received his long sustained efforts to maintain his theory ; we believe, however, that all reasonings, all intellectual processes of investigation should bend before the authority of facts which appear to us to be the necessary point of departure, and guide in anatomical researches.

### 3*d*. *The Doctrine of Conversion.*

The theory of conversion, as we have remarked, supposes the metamorphoses of the pulp into ivory or dentine. It differs from the doctrine of ossification in this, that it does not consider the tooth as a bone, but as an organ of a special nature, not foreign to the economy. M. Oudet, it is true, in defending the theory of secretion, states that the dental organ is possessed of a vital movement which allows it to be approximated to the other tissues; but he believes, in the present state of science, that the term *product of secretion* necessarily presupposes the idea of an inorganic body as deprived of life and submitted to the only physiological influences which govern dead matter. E. Geoffroy Saint Hilaire perfectly comprehended this necessity when he makes the tooth the product of crystallization, a true stalactite, entirely foreign to organic movement. Thus he logically defends an erroneous opinion.

Leeuwenhoeck, who wrote at Delft, in 1683, made the first attempt in favor of the theory which now occupies us. This writer, as is well known, was one of the first who made use of the microscope, and it is remarkable that at an epoch when the doctrine of secretion was not yet founded, the doctrine of conversion should have been produced ; thanks to the use of a powerful instrument, which, if it did not afford the complete solution of the problem in the hands of the Dutch observer, still established the fact that the tooth is not a bone, and did not permit the supposition that it was the result of secretion. Notwithstanding the efforts of Leeuwenhoeck, Rau,* departing from the way opened by his fellow countryman, established his doctrine without opposition and smothered the first useful efforts of his predecessor.

According to the anatomist of Delft,† the dental pulp is composed of a great number of blood vessels and other kinds of vessels, which afterwards become transformed into the dental canaliculi and serve also for the production and nutrition of the ivory. The canaliculi thus formed are directed from the centre of the tooth to the periphery and communicate with the vessels of the gum in order to form a perfect circulatory system, which will supply the dental organ. Yet this theory remained in oblivion until 1798, when Blake,‡ led by his researches to the ideas already broached by Leeuwenhoeck, suggested that a double molecular movement of composition and decomposition may exist in the ivory, which he substantiated by the observation of exostosis and other affections of this tissue. Thus

* *Disputatio Inauguralis ; Leyde*, 1694.

† *Philosophical Transactions*, 1683, p. 1002, and 1684, p. 568.

‡ *Disputatio de dentium formatione et Structuræ*, Edin., 1798.

he supposed that the tooth is supplied with a vascular system possessed of organic movement.

However, it must be confessed that the doctrine of conversion was really founded by two English anatomists, whose studies, dating back at least twenty years, have now received the more or less complete confirmation of a great number of writers. We speak of Nasmyth and Prof. Owen. From the appearance and spread of their works in Germany and England, micographers quickly declared themselves in favor of the new doctrine. But among this new generation of observers a division occurred, some maintained that the dental pulp was wholly metamorphosed into dentine; while on the other hand, it was supposed that this change only affected the superficial part of the organ. This distinction induces us to divide the study of this doctrine into two sub-divisions: the first comprehends the writers who believe in the total transformation of the germ—represented by Owen, Hannover, Tomes, Todd and Bowman, etc.; the second, those who are in favor of only partial transformation. This last division is defended by Nasmyth, Schwann, Kolliker, Lent, etc.

1st Division.—Richard Owen, in 1840,* was led to his theory by studying the production of the teeth in selacious (*chondropterygii*) fishes, in which the bulb produces the dentine by direct metamorphosis of its tissue. This peculiarity, already glanced at in 1837 by Muller,† induced Owen to consider the teeth of all animals as produced by an identical phenomenon.

According to him, the totality of the dental pulp is composed of nucleated cells suspended and united by a sub-granular plasma. The cells are more numerous about the super-

* Odontography, Lond., and art. *Teeth* in the Cyclopædia of Anat., t. iv, p. 864.

† Muller remarked, in myliobates and rhinoptères, that the dental pulp has the same size and form as the future tooth. The examination of its structure led him to observe the existence also of tubes of large diameter, receiving in their walls a kind of calcareous incrustation, which result in the final destruction of their calibre, and the total transformation of the bulb into ivory. (*Cours de Physiologie*, t. i, p. 319.)

ficial part of the organ, where they are disposed on the organ in a series of vertical lines. At this time the superficial cells increase by the division of their nuclei, the fragments of which become the centres of new cells. This whole mass is thus formed by the multiplication of a primitive cell which deposits in reciprocal parallel lines. The point of the cell touching the parietes atrophy, the nuclei become agglomerated, and thus form, by their union, continuous tubes, of which ulterior calcification produces the dentine and the canaliculi by which it is pervaded. As to the curves and anastomoses of the canaliculi, they are also determined, in this theory, by the curves which describe the anastomoses which unite the linear series of the dental cells.

According to Tomes,* whose point of view is analogous

* The translator here takes the liberty of quoting Mr. Tomes' own words:

"This creative process is beautifully illustrated in the development of the several parts of the dental organs. Thus, the papillæ springing up from the primary dental groove are principally composed of nucleated cells, cells capable of producing, by the growth of the contained nuclei, other cells similar in character and in purpose to those from which they have been formed. The dental papillæ increase in size by the formation of new cells from those already existing. In a short time the papillæ are contained in follicles; still although much increased in size, they are mainly composed of nucleated cells connected by a homogeneous transparent thick fluid, a blastema or plasma. In the base of the papillæ, when inclosed in follicles, blood vessels are formed, they, however, are not immediately concerned in the cellular development, but probably by transudation through their coats, furnish the homogeneous plasma which is immediately concerned. In the process of time, the precise period varying in the different teeth, the follicles become sacs.

"The papillæ, which is now designated the pulp, assumes the shape of the future tooth, and commences to fulfil the ultimate purpose of its existence, in the formation of dentine. Previous to the development of tooth-substance, the inner surface of the sac becomes separated from the surface of the pulp, the intervening space being occupied by a soft gelatinous granular matter. This is the formative pulp for the development of the enamel. At this stage of dental formation, we have a closed sac containing two formative pulps, one for the development of the dentine, the other for the formation of the enamel; the former in the shape of the crown of the future tooth, the latter forming a cap over the dentinal pulp. At a later period we have a matrix for the formation of the cementum.

"The dentinal pulp, from its earliest appearance to the time of its transition into dentine, is mainly composed of a series of nucleated cells, united and supported by plasma, is supplied with vessels, and during the greater part of its existence, with nerves."

to that of Prof. Owen, the dental pulp is formed of nucleated cells disposed in the midst of a plasma pervaded by vessels and nerves ; this plasma is also equally pervaded in all its parts by an alveolar web, in the meshes of which the cells of dentine are received.

These cells, composed of proper membranous walls and a central cavity represented by the nucleus, are primitively dispersed in the mass of the organ, yet gradually they range end to end, following reciprocal parallel lines, and constitute what Tomes calls the *linear disposition* of the dental cells.

In this state, the cell elongates as well as the nucleus or cavity which it contains ; its proper wall then disappears, and the nuclei uniting by their extremities, form a complete tube, the walls of which are formed by the same substance as the cell. The linear series of neighboring cells then unite to the first, and form the anastomoses and ramifications which we find in the canaliculi. At this moment the vessels of the pulp carry the calcareous materials, which give to this cellular mass the consistence and character of dentine, pervaded by ramifying canaliculi. This phenomenon takes place, in the first place, in the superficial parts of the pulp, but soon goes on to the whole mass which thus becomes completely metamorphosed.

Lastly, Hannover, who has adopted and defended the doctrine of Owen, has recently published at Copenhagen, a remarkable monograph on the development and structure of the teeth.* The influence of the author, and the seduc-

In addition to the elements, there is one other that pertains to the pulp, prior to the commencement of those changes which prepare for its conversion into dentine. This element is a fine alveolar tissue, a meshwork of delicate fibres and bands of homogeneous matter, in the thicker part of the walls of which are scattered here and there nucleated cells, ; while the meshes are occupied by a thick, clear, homogeneous fluid or plasma ; and in this are a number of nucleated cells." A Course of Lectures on Dental Physiology and Surgery, lecture 5, Lond.

* Entwickelung und den Bau des Saügethierzahns ; Aus den Verhandlungen der kaiserl. Leopold Carolinischen Akademie der Naturforscher, t. xxv, p. 11.

tive aspect under which this work has been presented, induces us to offer some succinct remarks upon it.

Hannover says that the human follicle, before the beginning of dentification, is composed of four principal parts: 1st, The germ of the dentine, placed at the bottom of the dental sac; 2d, The germ of the enamel, placed like a cap above the preceding, and composed exclusively of cells, (enamel cells, adamantine membrane, of some writers;) 3d, The germ of the cement of stellated and ramified cells (enamel germ) and making a complete envelop of all of the two preceding germs except their base; the transformation of this germ into cement, takes place by simple ossification; 4th, The *membrana intermedia*, a transparent and amorphous membrane, separating the germ of cement from the two other germs of enamel and dentine, so that by its external face it is in contact with the first, whilst the internal covers the two others.

The dentine cells are placed in the midst of amorphous matter without a proper envelop, and are disposed in superimposed layers. These cells, provided with nuclei, are primitively spherical, and are ranged parallel to one another and perpendicular to the surface of the germ. At this stage, the proper parietes of the cell and the nucleus elongate simultaneously, and from long prolongations, which ramifying, anastomosing with the prolongations of the neighboring cells, thus become dental canaliculi; the envelop of these forms their wall, the nucleus forms the cavity (*lumen*) of the canaliculi. As to the intertubular substance it is nothing more than the amorphous matter which separates the cells. In this condition, the canaliculi and the matter which separates them become impregnated with calcareous matter, and dentification is terminated.

We would remark, in reference to this theory, that the germ of the cement is nothing else than the enamel germ of all the authors, whilst this last organ cannot but be composed by the cells of enamel or the adamantine membrane; moreover, the presence of the *membrana intermedia* seems to intervene for the necessities of the description; for no anatomist has yet noticed such a membrane so situated. Its existence, it is true, renders the intelligence of the development very simple and very complete; the enamel, as we shall see, is covered by a thin amorphous pellicle, according to Hannover, nothing more than his pretended *membrana intermedia,* subjacent to the enamel. Moreover, we will find that the cement germ of Hannover (enamel germ of writers) follows, by its development, the production of the enamel, and that when this substance is produced, it atrophies and disappears, a circumstance which betrays a certain connection between them; we think then we cannot attribute the formation of the cement to one organ, the disappearance of which coincides with the achievement of the crown and the beginning of cementous production.

In that which concerns the formation of ivory, Hannover's theory is analogous to that of Owen and Tomes. We would answer to all of these authors, that it is impossible to the totality of the pulp as constituted by the cells of ivory; the most simple inspection offers the proof; and further, it cannot be admitted that the nucleus forms the cavity of the canaliculus, since the nucleus is a solid body, and we are unable to find it isolated and broken into fragments. Again—and this argument is particularly addressed to Hannover—the prolongations, which are sometimes provided with cells of enamel, cannot be regarded as canaliculi, since they are directed towards the pulp—not towards the ivory, that is to say, inversely to the direction of the canaliculi.

2d Division. The researches of Nasmyth, dating from 1839, have powerfully agreed with those of Owen, which they preceded by several months, to the ruin of ancient doctrines in England.*

*** Researches on the Development, Structure and Diseases of the Teeth. Lond. 1839.**

This writer describes the dental pulp as an organ formed of two principal parts, a central vascular and a superficial, composed of a very considerable number of very minute cells, which, by their arrangement, similate the reticulated disproportion of the skeleton of a cuttle-fish leaf. (*loc. cit.* p. 42.) It is this part of the pulp thus constituted, which calcifies, to form dentine; its structure is there purely alveolar, that they may thus give birth to the tissue. The dental pulp and the ivory are consequently the same substance; there it is a cellular reticulated web, here the same web hardened by the calcareous elements which the vessels of the lower part exude from their parietes.

It seems, after this, that Nasmyth has completely ignored the dentine cells which occupy the superficial layers of the pulp, and that which he describes as a reticulated cellular disposition, is nothing more than the fibro-plastic bodies, forming the mass of the organ, and destined, according to this writer, to become calcified and form ivory. This last substance, consequently, is constituted by an alveolar tissue, composed of fibres, offering furrows like a string of beads, completely deprived of canaliculi and placed in organic calcified matter, in the midst of which it is not possible to recognize the existence of primitive organic elements.

What is most surprising in these errors of the distinguished anatomist, whom we have here cited, is, that he seems to have employed the means of investigation which have conducted others to a different conclusion. It must be admitted, for example, that Nasmyth has been governed by certain speculative ideas, to refuse to see canaliculi in the ivory, when no one else has denied their existence.

Schwann* says that the superficial layers of the pulp are constituted by an assemblage of cylindrioal cells; and the lower part is composed of small, rounded and nucleated cells. The cylindrical cells gradually elongate, after the

***Mikroskopische Untersuchungen reber die Uebereinstimmung in der Structur und den Wachsthum de der Thiere und Pflanzen; Berlin, 1839.**

fashion of fibres, and become impregnated with calcareous matter, while the vessels retire towards the centre.

In this opinion we perceive that the dental pulp presents a decided fibrous disposition, and the ivory is made by the calcification of these fibres.

Kolliker* and Lent† have taken and developed the researches of Schwann in a more precise manner. According to them the dental pulp is composed of two very distinct parts: the one central, vascular and an entire stranger itself to dentification; the other superficial and composed of special cells, which, by their successive transformations, constitute the ivory. The cells of dentine are the only elements concerned in the formation of ivory, and to Lent should we attribute the positive determination of the operation of this transformation. Upon the teeth in process of development, and nearly reduced to a homogeneous mass by prolonged maceration in hydrochloric acid, Lent, and afterwards Kolliker, were able to isolate little masses, provided with more or less ramifying prolongations.—These masses are only the ivory cells furnished with their canaliculi. We have, indeed, often met with cells in mass, furnished with ordinary simple prolongations, sometimes in the horse and sheep, bifurcated; but this disposition, which besides, is far from being constant, seems to us to be entirely foreign to the formation of canaliculi, since these have an inverse direction from those of the cellular prolongations, which for the rest are not tubulated.

Be that as it may, these two writers found upon this simple fact, their whole theory of the formation of ivory, and which may be summed up in the following terms:

1st. The dental canaliculi are true prolongations of the cells of ivory, prolongations which give birth to secondary branches by which they communicate (Kolliker, *Histologie Humaine*, p. 201.) And in the majority of cases a simple

* Histologie Humaine, p. 434, and Mikrokopische Anatomie, t. 11, p. 97; 1852.

† Beiträge Zur Entwickel, d. Zahnbeiner und Schmelzes, in Zeitchr. F. Wiss. Zool., vi, Cah.

cell suffices to produce an entire canaliculus, or at least the greater portion of one.

2d. The fundamental substance of ivory does not provide ivory cells; resembling intercellular substances, it results either from an exudation of these cells, or from the dental germ. (Kolliker, *loc. cit.*, p. 435.)

Thus then, according to the opinion of these German anatomists, the dentine results from a transformation of only a part of the histological elements of the germ, the superficial part composed of cells: the central part does not undergo dentification in some species of animals, and perhaps is exceptional in man, where this phenomenon has been established by the observations of vascular ivory by Tomes and Owen, (vaso-dentine.) In this case, adds Kolliker, the vascular ivory affects a great resemblance to osseous tissue, and it is far removed from the ordinary structure of ivory.

We here perceive that the doctrine of conversion reposes upon this fact, that the dental pulp intervenes directly, by its proper histological elements in the production of dentine, and one readily recognizes that most authors that defend it are far from assimilating the tooth to a bone. Kolliker, however, following the researches of Lent, and his discovery of prolongations to the dental cells, finds a certain analogy of development between the tooth and bone; for the cells of cartilage are also surrounded by tubular prolongations in order to form the osseous corpuscles and their canaliculi. This resemblance is seducing, no doubt, but we should not forget that the prolongations of dental cells are not tubular.

### 4th. *Doctrine of Deposition.*

### RASCHKOW and HUXLEY.

This doctrine, contemporary of the preceding, is due to Raschkow, (Breslau, 1835,) and has been adopted with some modifications by Huxley in England. It differs from the preceding doctrine in the respect that the histological elements of the germ do not concur directly in the formation of the dental substance, which is *deposited*, layer by layer, between the germ and the *membrana præformativa*, which serves as an envelop, whilst this organ undergoes a gradual recession, a progressive atrophy in proportion as it gives birth to the elements of ivory.

According to Raschkow,* the dentine or ivory is composed of fibres placed under the *membrana præformativa*, which undergo calcification at a later period. These fibres, thus disposed, gradually harden by the assimilation of calcareous elements furnished by the pulp, and constitute the covering of ivory, while the canaliculi, the disposition of which Raschkow seems to have determined, result from intervals formed between these fibres. As to the pulp, it undergoes progressive absorption in proportion as it deposits the dental substance. Such is the theory of this writer, which we find in the following paragraph:

"Postquam . . . fibrarum dentalium stratum depositum est, idem processus continuo ab externa regione internam versus progreditur germinis dentalis parenchymate materiam suppeditante. . . . . Conversœ fibrarum dentalium flexuræ quæ juxta latitudinis dimensionem crescunt dum ab externa regione internam versus procedunt sibi invicem appositæ continuos canaliculos effingunt, qui ad substantiæ dentalis peripheriam exorsi multis porvis anfractibus ad pulpam dentalem cavumque ipsius tendunt; ibique aperti finiuntur novis ibi quamdiu substantiæ dentalis formatio durat fibris dentalibus aggregandis inservientes."†

This doctrine, forgetting, or misconceiving the body of contemporary authors, seems to have been adopted by Huxley in England, who has developed it under the name of the *Doctrine* of *Deposition*. This writer‡ has, among

* *Meletemata circa Dentium Mammalium Evolutionem.* Vratislav, 1835.

† For a full exposition of the views promulgated by Raschkow, see Nasmyth's "Historical Introduction," published in vol. iii, of the Am. Library of Dental Science, 1843.—*Tr.*

‡ *On the Development of the Teeth and on the Nature and Import of Nasmyth's Persistent Capsule.* [Quart. Jour. of Microscopical Science, April, 1853.]

other things, advanced certain peculiar ideas in regard to the development of the teeth, which we should here reply to. According to him the dental pulp is invested primitively by a proper membrane—*basement membrane*—(*membrana præformativa,*) under which all the dental tissues are formed, enamel, ivory, cement. The pulp is composed of very small, rounded, oval or polyhedral cells, containing granulations. They are found in the midst of a transparent amorphous substance, which does not contribute more than the cells to the formation of the ivory; for this substance, if we may believe this writer, deposits molecule after molecule between the dental germ and the *membrana præformativa,* so that, in this theory, the histological elements of the germ do not contribute further to the formation of the tooth. This opinion differs from the preceding in this respect; it rejects the existence of fibres in the ivory, but admits the presence of canaliculi, which Huxley considers as lacuni hollowed into the thickness of the substance during the work of development.

Such are the doctrines which have been presented on the development of ivory. In the midst of this conflict of opinions, so different and often contradictory, how shall we decide? Our description of the development of ivory already answers the question; nevertheless, we shall now proceed to develop and establish our theory.

We think that the elements concerned in the formation of ivory, that is to say, the dentine cells are spontaneously produced by *genesis* or *spontaneous generation* at the surface of the dental pulp, which furnishes the material necessary to this production. These cells are formed in rows superimposed, one on the other, a disposition which we find in the concentric layers of which the ivory is formed. Thus constituted, each of these cells represents a living individual, a complete organism, possessing all the properties belonging to the anatomical elements of the class of products of perfection,* that is, the vegetative properties of nutrition, of development and genesis. Once developed, the cells undergo certain modifications resulting from the organic movement, which is accomplished within them; their nucleus disappears either by absorption or by the invasion of the calcareous material, and the new row of cells develop and unite to those previously formed, the body of the new cell corresponding to the body of the old cell, and the intervals equally corresponding in order to continue the direction of the canaliculi.

* It is important here to give an explanation of the word *product*, created by de Blainville, and the sense of which it is well to define. The term is used here only in the anatomical sense, for in a physiological point of view there is no such thing as birth, generation, of the products completely formed, but simply the generation of a blastema serving for the genesis of products. This blastema may itself, present differences. According to M. Robin, it may be either real or apparent: real when we may follow the development of the anatomical elements in its midst, operating directly at the expense of its own substance; virtual or apparent, when in proportion as it is produced it is immediately replaced, molecule by molecule, by the elements it is called upon to constitute; such is the case in ivory. Below we give some developments on products in general.

"In the different order of parts which compose the organism, some are accessory at the side of others, as to the mass and as to the passivity of the acts which they accomplish, which have nothing directly essential, and do not serve to favor and perfect the acts of the other. We may call the first products, and the second, constituents. The products are never placed, except for a more or less limited period upon the whole of the surfaces, as much internal as well as external with those with which they are contiguous, without contracting any actual continuity; whether they are liquids, semi-liquids, etc., and are contained in the reservoirs communicating to the exterior, and to the secreting organs. Among the products, there are those among them, such as the saliva, the gastric juice, the ovale, the hair, the nails, etc., which are the products of improvement; these are solid products, narrowly united by a true tissue in the structure of certain apparatus to which they furnish essential means of improvement." (Ch. Robin Dictionn. de Nysten, 10th edit., 1856, art. *Produit.*)

"A character inherent to products considered in reference to their nutrition, is the stability of their combinations. The principals which compose them are firmly fixed and combined, and are separated with difficulty. It is necessary to employ powerful acids to raise successively the principles which are added to the part formed in such anatomical element. Instability, on the contrary, constitutes the predominant character of the constituents." (Ch. Robin, *Anat. Generale.*)

