# Errata

**Published:** 1859-10

**Authors:** 


303, line 7 from the top, insert after "*autogenesis*," a semicolon, and the word *thus.*

303, line 5 from the bottom, insert a *t* after *f*, in *often.*

304, " 22 from the top, for *admitted*, read *states.*

304, " 3 from bottom, for *should*, read *were destined to.*

305, " 12 from top, strike out *and*, and read *starts*, for *starting ;* also insert *and* before *concludes.*

305, " 7 " bottom, insert *the* after *of.*

305, " 6 " " for *remain with*, read *devolve upon.*

306, " 4 " top, strike out *however.*

308, insert quotation marks at the end of the first paragraph.

309, line 3 from top, strike out *by*, and *for him.*

309, " 15 " bottom, for *a stranger*, read *foreign.*

309, " 10 " " insert a comma after *observation.*

309, " 9 " " " " " *them.*

310, " 5 " top " *that*, before *the.*

311, " 1 " " for *would be*, read *is.*

311, " 13 " " for *that which*, read *as far as it.*

311, " 18 " " for *are*, read *is.*

311, " 22 " " for *in a*, read *of the.*

311, note ; strike out second *u* in *humanuorum.*

313, strike out the comma in the last line of first paragraph.

319, line 7 from top, insert *which is*, after *pellicle ;* strike out *according to ;* read *Hannover's* for *Hannover ;* and strike out, *nothing more than is.*

319, line 16 from top, for *one*, read *an.*

319, " 22 " " insert *consider* after *to.*

320, " 8 " " strike out *that they may thus give birth to*, and read *as also ;* after *tissue*, read, *to which it gives birth.*

321, line 6 from top, for *taken*, read *adopted.*

Page 321, line 9 from top, for *an entire stranger*, read *of.*
321, " 10 " " insert *entirely foreign*, after *self.*
321, " 18 " " for *were*, read *was.*
321, " 20 " " for *only*, read *simply.*
321, " 6 " bottom, strike out the last comma.
321, " 5 " " " " *and.*
325, " 18 " " in note. For *which*, read *others;* and for *do not*, read *only.*
325, line 12 from bottom, after *and*, read *annexed.*
325, " 9 " " for *narrowly*, read *closely.*
325, " 2 " " for *such*, read *each.*

